# Supplementary material for: A role for vitamin D and omega-3 fatty acids in major depression? An exploration using genomics
Source: Transl Psychiatry. 2019 Sep 5;9:219. doi: 10.1038/s41398-019-0554-y (PMC6728377; doi:10.1038/s41398-019-0554-y)
Supplement: Supplementary file 1 — Supplemental Materials [file 41398_2019_554_MOESM1_ESM.docx]

**SUPPLEMENTAL MATERIALS**

***s1. Genotyping, quality control and imputation*** pag.2

***s2.Processing of n3-PUFA GWAS summary statistics*** pag.3

***Supplemental eTable 1*** pag.3

***s3. PRS analyses*** pag.4

***s3.1 PRS generation*** pag.4

***s3.2 Estimation of variance explained by the PRS*** pag.4

***s4. Mendelian randomization*** pag.6

***s4.1 Selection of independent SNPs as instruments for MR*** pag.6

***s4.2 Power to detect a significant causal estimate in one-sample MR analyses*** pag.7

***s4.3 MR plots***  pag.8

***Supplemental eFigure 1*** pag.8

***Supplemental eFigure 2*** pag.9

***Supplemental eFigure 3*** pag.10

***Supplemental eFigure 4*** pag.11

***s4.4 PheWAS for rs174546*** pag.12

***Supplemental eFigure 5*** pag.12

***s4.5 MR-Egger*** pag.13

***Supplemental eTable 2*** pag.13

***Supplemental eTable 3*** pag.14

***s5. Analyses based on quantitative genetics theory*** pag.15

***Supplemental eTable 4*** pag.16

***References*** pag.18

**Data and software availability** pag.21

***s1. Genotyping, quality control and imputation***

Genotyping, quality control and imputation were previously described in details^1^. Briefly, 95% of the samples were genotyped on the Affymetrix 6.0 Human SNP array and the remaining on the Perlegen-Affymetrix 5.0 array. After platform-specific QC the missing SNP genotypes between each platform were imputed using the GONL (Genome of the Netherlands)^2^ reference panel and then merged. Following more stringent QC, the SNPs from this cross-platform GONL imputed dataset (~1.2M) were used for a second round of imputations to the 1000G Phase 3 all ancestries reference panel using the Michigan Imputation Server^3^. Genotype data were used to build a relationship matrix measuring genetic similarity using GCTA^4^ , which was pruned at 0.05 threshold in order to reatain unrelated participants.

***s2. Processing of n3-PUFA GWAS summary statistics***

Summary statistics for n3-PUFA were obtained from the MAGNETIC NMR GWAS^5^ examining the same metabolomics platform (Nightingale Health Ltd., Helsinki, Finland) adopted in NESDA on up to 24925 individuals. The original manuscript reports only global patterns of results across several metabolites. We processed the specific summary statistics for n3-PUFA in order to identify independent genome-wide significant SNPs by using the FUMA tool^6^. We applied the SNP2GENE function using GWAS summary statistics as an input and providing annotation of genomic areas identified by lead SNPs. The following criteria were applied: SNPs with MAF ≥ 0.01 were retained, the MHC region was excluded from annotation (due to long-range LD), an r^2^ 0.1 threshold was used to define LD structure of the lead SNP, maximum distance between LD blocks was set at 500kb and the EUR population of 1000Genomes was used as LD reference. Seven independent genome-wide significant SNPs from 5 genomic loci were identified.

**Supplemtal eTable 1** Independent genome-wide significant SNPs in n3-PUFA GWAS

| **rsid** | **chr** | **position** | ***p*** |
| --- | --- | --- | --- |
| rs174546 | 11 | 61569830 | 1.2E-34 |
| rs1260326 | 2 | 27730940 | 3.4E-14 |
| rs143988316 | 19 | 19667254 | 3.0E-12 |
| rs11604424 | 11 | 116651115 | 3.3E-10 |
| rs145717049 | 19 | 19130096 | 6.7E-09 |
| rs150617279 | 19 | 20139234 | 7.1E-09 |

The top SNP was located in the 3'-UTR region of FADS1 (fatty acid desaturase enzyme, involved in fatty acids metabolism), confirming the biological relevance of the top GWAS hits for this trait. Consistently, other significant loci included relevant genes for hepatic lipid metabolisms, such as LIPC (lipase C) and GCKR (Glucokinase regulatory protein).

***s3. PRS analyses***

***s3.1 PRS generation***

Polygenic risk scores (PRS) were calculated as the number of risk alleles weighted by their effect sizes from the relative discovery GWAS summary statistics using PLINK.^7^

PRS for 25-OH-D and n3-PUFA were based on genome-wide significant SNPs, harboring relevant genes for the synthesis and metabolism of these markers. For 25-OH-D we selected the 6 independent SNPs reported in the discovery GWAS^8^. For N-3 PUFAs, we identified 7 independent genome-wide significant SNPs by processing GWAS summary statistics (supplemental materials s2). In NESDA, the SNPs were selected in the 1000Genomes imputed set (see s1 section).

PRS for MDD was built based on the full polygenic signal from the MDD GWAS.^9^ Since NESDA data were part of the GWAS, we re-ran the MDD meta-analyses after removal of overlapping datasets (~3K samples). Summary statistics of the discovery were filtered by removing In/Del and strand ambiguous variants, SNPs with INFO < 0.9, MAF < 0.01. Overlapping SNPs between the ~1.2M cross-platform GONL imputed (see s1 section) and those retained from the discovery summary statistics were carried forward. PRS were built according to LDpred method^10^ using the dedicated software. 1000 unrelated individuals were selected to calculate LD for reference. The fraction of causal SNPs was set at 5% consistently with previous analyses.^11^

***s3.2 Estimation of variance explained by the PRS***

Same- and cross-trait associations of the different PRS with 25-OH-D and n3-PUFA concentrations and with MDD diagnosis were estimated using regression models (linear for 25-OH-D and n3-PUFA and binary logistic for MDD) adjusted for sex and 10 ancestry-informative genetic principal components. In analyses with 25-OH-D or n3-PUFA as outcomes, the proportion of variance explained by the PRS was estimated based in the difference in R^2^ between a linear model including only covariates and a model additionally including the PRS. In analyses with MDD as outcome, Nagelkerke’s pseudo-R^2^ was derived and corrected for the covariates by substituting the null model in Nagelkerke’s equation for the model including the covariates. The corrected pseudo-R^2^ obtained was then re-scaled to the liability scale according to Lee et al.,^12^ obtaining a value directly comparable with heritability and robust against ascertainment bias. Linear transformation on the liability scale was based on lifetime risk (K) for MDD of 0.15.

***s4. Mendelian randomization***

***s4.1 Selection of independent SNPs as instruments for MR***

Two-sample Mendelian randomization (2SMR) analyses^13^ based on GWAS summary statistics were performed to test the potential causal role of 25-OH-D and n3-PUFA on MDD risk and, inversely, of MDD on 25-OH-D and n3-PUFA levels. For each trait used as exposure, genome-wide significant independent SNPs were selected as instruments.

For analyses focusing on 25-OH-D as exposure, the 6 independent genome-wide significant SNPs reported in the discovery GWAS^8^ were used. For analyses focusing on n3-PUFA as exposure, the 7 independent genome-wide significant SNPs (see s2 section) were selected. However, since rs145717049 was not present in summary statistics of MDD GWAS^9^ we replaced it with its best LD proxy (r^2^ = 0.3, from LDlink 3.3.0) in EUR population of 1000Genomes.

For MR analyses focusing on MDD as exposure and 25-OH-D as outcome, we selected 37 independent SNPs from the PGC MDD GWAS. In order to maximize the number of overlapping variants across the two GWAS with a different number of interrogated SNPs (due to difference in imputation reference panel: 1000 Genomes for MDD and HapMap2 for 25-OH-D) we performed the following selection steps: firstly, we identified 873 non strand-ambiguous SNPs (with *p* < 5.0e-8 in the MDD GWAS) available in both GWAS; we then retained only one SNP from the extended MHC region (due to long-range LD) and the rest was clumped using PLINK v1.9 using a 500kb window and an r^2^ 0.1 with the EUR population of 1000Genomes as LD reference.

We applied the same selection steps for MR analyses focusing on MDD as exposure and ne-PUFA as outcome and we selected 44 independent SNPs from the PGC MDD GWAS to be used as instruments.

***s4.2 Power to detect a significant causal estimate in one-sample MR analyses***

The use of 2SMR allows to increase substantially the power of genetic instruments by leveraging on effect sizes estimated in large sample size of GWAS, as compared to individual-level data MR that has usually low power. For instance, even with a strong instrument as the PRS for increased 25-OH-D used hereby in NESDA data (3.5% of variance explained in 25-OH-D levels, corresponding to an F-statistic of 89.93, with F > 10 usually considered an adequate instrument) 80% statistical power to detect a significant (at α=0.05) causal effect of 25-OH-D on lifetime MDD (exposure-outcome association unadjusted OR=0.79) could be reached with a sample size of 16069 (half cases) (from mRnd, power calculator for MR^12^). Similarly, using the PRS for n3-PUFA (0.8% variance explained, F-statistic 26.85) 80% statistical power to detect a significant causal effect of n3-PUFA on MDD (exposure-outcome association unadjusted OR=0.87) could be reached with a sample size of 202028 (half cases).

***s4.3MR plots***

Panels: A. 2SMR estimates; B. Single SNP analyses ; C. Leave-one-out analyses

Units of measure in discovery GWAS: 25-OH-D, 1 unit increase in (log) concentrations; n3-PUFA, 1 unit increase in (﻿inverse normal transformation) concentrations; MDD, 1 log-unit increase in risk.

***Supplemtal eFigure 1.*** 2SMR analyses estimating causal effects of 25-OH-D on MDD

***Supplemtal eFigure 2.*** 2SMR analyses estimating causal effects of n3-PUFA on MDD

***Supplemtal eFigure 3.*** 2SMR analyses estimating causal effects of MDD on 25-OH-D

******

***Supplemtal eFigure 4.*** 2SMR analyses estimating causal effects of MDD on n3-PUFA

******

***s4.4PheWAS for rs174546***

In 2SMR analyses testing the causal effect of n3-PUFA on MDD we identified the SNP rs174546 as potential source of heterogeneity. In order to evaluate whether this heterogeneity was indicating potential horizontal pleiotropy (the instrument/SNP is associated to the outcome trough pathways others than the exposure), we performed a PheWAS (phenome-wide association scan) using the GWAS ATLAS Resource^14^. 3,798 traits were scanned and 67 significant (0.05/3,798 = 1.3e-05) associations were retrieved, including a wide array of traits.

***Supplemtal eFigure 5.*** Significant associations of rs174546 with traits organized in major domains

***
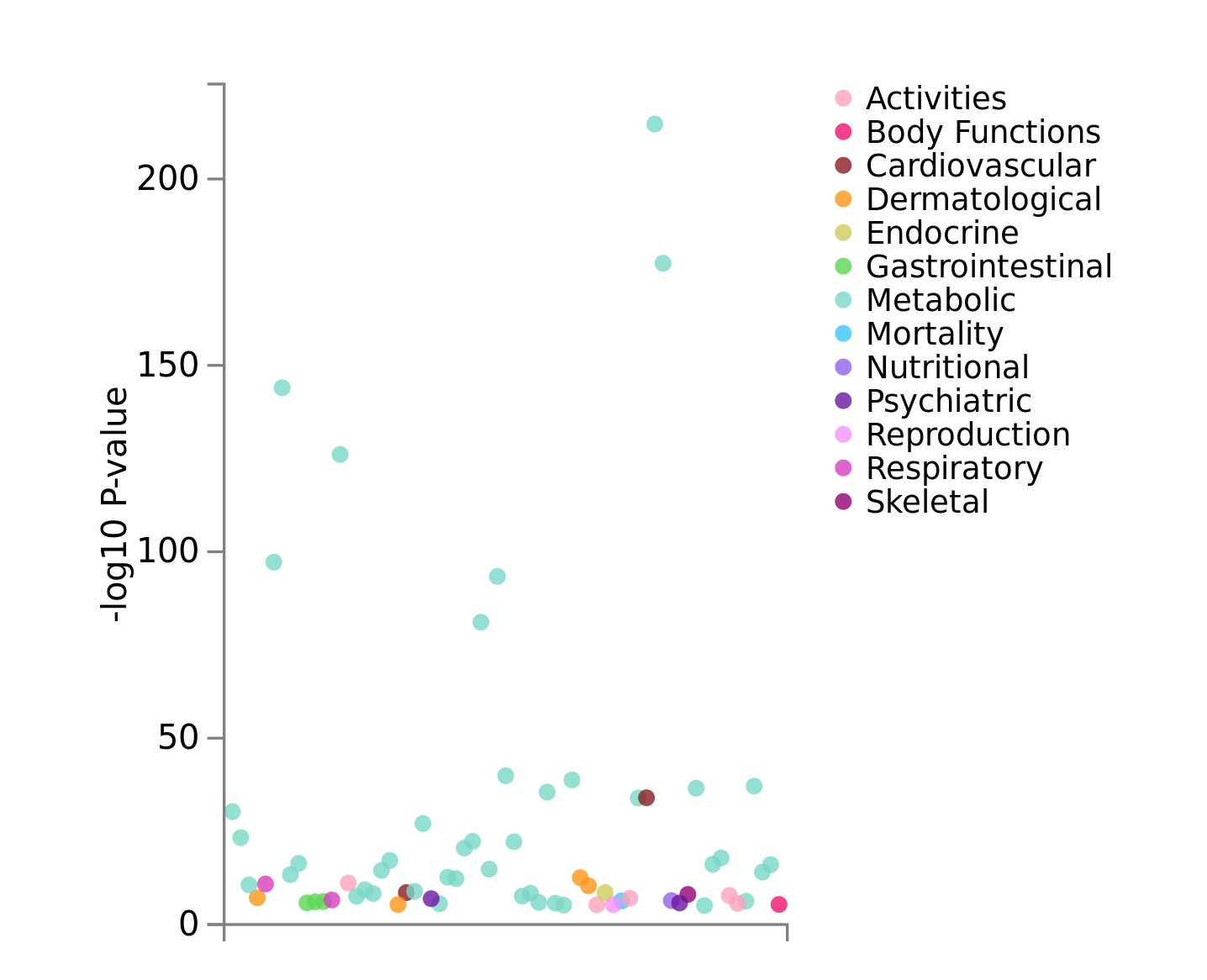
***

***s4.5MR-Egger***

Additional analyses were performed using the MR-Egger method^15^ relying on the InSIDE assumption (the magnitude of any pleiotropic effects should not correlate with the magnitude of the main effect) less conservative as the lack of horizontal pleiotropy The number of instruments used to test the causal effect of 25-OH-D and n3-PUFA was lower than that (N=10) recommended^15^ to run an adequately powered MR-Egger analyses. The estimates were substantially consistent with those obtained by other MR analyses. Furthermore, estimates from MR-Egger intercepts suggests the lack of substantial pleiotropy in analyses

***Supplemtal eTable 2.*** 2SMR analyses using the MR-Egger method

|  |  |  |  | **MR-Egger** | | | | | | |
| --- | --- | --- | --- | --- | --- | --- | --- | --- | --- | --- |
|  |  |  |  | **Intercept** | | |  | **Slope** | | |
| **Exposure** | **Outcome** | **N SNPs** |  | **estimate** | **SE** | ***p*** |  | **estimate** | **SE** | ***p*** |
|  |  |  |  |  |  |  |  |  |  |  |
| **25-OH-D** | **MDD** | **6** |  | 0.004 | 0.01 | 0.74 |  | -0.03 | 0.09 | 0.71 |
|  |  |  |  |  |  |  |  |  |  |  |
| **n3-PUFA** | **MDD** | **7** |  | 0.01 | 0.02 | 0.71 |  | -0.12 | 0.16 | 0.50 |
|  |  |  |  |  |  |  |  |  |  |  |
| **MDD** | **25-OH-D** | **37** |  | 0.003 | 0.003 | 0.29 |  | -0.11 | 0.09 | 0.23 |
|  |  |  |  |  |  |  |  |  |  |  |
| **MDD** | **n3-PUFA** | **44** |  | 0.004 | 0.011 | 0.74 |  | -0.079 | 0.337 | 0.82 |
|  |  |  |  |  |  |  |  |  |  |  |

***Supplemtal eTable 3.*** 2SMR analyses using MR-Egger and MR-RAPS methods with polygenic instruments including larger sets of SNPs.

|  |  |  |  | **MR-Egger** | | |  | **MR-RAPS** | | |
| --- | --- | --- | --- | --- | --- | --- | --- | --- | --- | --- |
| **Exposure** | **Outcome** | **N SNPs** |  | **estimate** | **SE** | ***p*** |  | **estimate** | **SE** | ***p*** |
|  |  |  |  |  |  |  |  |  |  |  |
| **25-OH-D** | **MDD** | **132** |  | -0.01 | 0.06 | 0.90 |  | -0.02 | 0.04 | 0.64 |
|  |  |  |  |  |  |  |  |  |  |  |
| **n3-PUFAs*^a^*** | **MDD** | **132** |  | 0.03 | 0.02 | 0.12 |  | -0.01 | 0.01 | 0.11 |
|  |  |  |  |  |  |  |  |  |  |  |
| **MDD** | **25-OH-D** | **477** |  | 0.01 | 0.02 | 0.56 |  | -0.01 | 0.01 | 0.13 |
|  |  |  |  |  |  |  |  |  |  |  |
| **MDD** | **n3-PUFAs** | **675** |  | 0.003 | 0.058 | 0.96 |  | -0.02 | 0.024 | 0.46 |
|  |  |  |  |  |  |  |  |  |  |  |

RAPS analyses performed with default settings in MR-Base R package

***^a^*** Potential directional horizontal pleiotropy indicated by MR-Egger intercept: estimate= -0.005, se=0.002, *p*=1.8e-3

***s5. Analyses based on quantitative genetics theory***

MR leverages on pleiotropic effects of SNPs on both the exposure (25-OH-D or n3-PUFA) and the outcome (MDD), in particular vertical pleiotropy emerging from the fact that the SNPs used as instruments are associated with the outcome only through the exposure. It could be hypothesize that, provided an existing causal exposure-outcome relationship, SNPs significantly associated with the exposure would be associated also with the outcome in an adequately powered GWAS. Using quantitative genetics theory the expected power, assuming a causal relationship, to detect a significant association in the MDD GWAS^9^ for SNPs genome-wide significantly related to 25-OH-D or n3-PUFA in the discovery GWAS^5,8^ could be estimated. These power estimations can be compared to the empirical findings from the GWAS on MDD, to explore if 25-OH-D or n3-PUFA may be causal for MDD. In other words, when the estimated power is large assuming a causal impact of 25-OH-D or n3-PUFA on MDD, but when SNPs are not significantly linked to MDD, this would suggest that 25-OH-D or n3-PUFA are not causal for MDD.

The SNP effects on Vitamin D found in the GWAS^8^ were scaled to standardized SNP effects (effects when 25-OH-D would have been scaled to a variance of 1 for the GWAS analyses). For this, the equation from Okbay et al.^16^ was used $standardize beta=(beta/SE)/\sqrt{N*2*AF(1-AF)}$ with $N=79,366$ and $AF$ the allele frequency as displayed in eTable 3. Subsequently, the variance explained in 25-OH-D was estimated as ${Var}_{SNP\to VitD}={(standardize beta)}^{2}*2*AF*(1-AF)$. The combined impact of the six SNPs thus adds up to 2.7% variance explained in 25-OH-D, which is very close to the combined impact presented by Jiang et al^8^ of 2.8%, indicating our approximation of the variance explained in Vitamin D is correct.

The variance explained in MDD liability by 25-OH-D was estimated at 5%, and by n3-PUFA at 4% based on Lee et al^12^ method. When the link between 25-OH-D and MDD would be causal, SNPs would thus explain ${{Var}_{SNP\to MDD}=0.05*Var}_{SNP\to exposure}$ of variance in MDD. From this the power to detect an association between the SNP and MDD in the related GWAS (135,458 cases 344,901 controls) was approximated as described previously.^17^ In a second set of analyses, we assumed 25% of the link between the exposure and MDD would be causal, with 25-OH-D thus explaining 1.25% of variance in MDD and n3-PUFA 0.1%. rs174546 was excluded from analyses of n3-PUFA, because it showed potential horizontal pleiotropy (supplemental materials s4.3).

The power to detect SNP-effects in MDD GWAS was approximated at 0.74-1.00 and 0.13-1.00 assuming, respectively, a full and quarter of causal link between 25-OH-D and MDD, and at 0.4-0.87 and 0.13-0.34 assuming, respectively, a full and a quarter of causal link between n3-PUFA and MDD. Thus, when 25-OH-D or n3-PUFA would be causal for MDD, some of the SNPs would be expected to be associated with MDD. However, in MDD GWAS, none of the SNPs is associated with MDD at p<0.05. Note that although rs150617279 has a significant impact on MDD, this is in the opposite direction as would be expected under causality of n3-PUFA on MDD (n3-PUFA negative correlated to MDD, so the SNP should have an opposing direction of effect when the association would be causal).

In conclusion, these results strongly suggest that the phenotypic link between 25-OH-D and MDD and between n3-PUFA and MDD is not attributable to a causal effects.

***Supplemtal eTable 4.***  Estimated power, assuming a causal relationship, to detect a significant association in the MDD GWAS for SNPs genome-wide significantly related to 25-OH-D or n3-PUFA.

|  | | | | | | | | | | | | |
| --- | --- | --- | --- | --- | --- | --- | --- | --- | --- | --- | --- | --- |
|  |  | SNP effect on exposure | |  | Power to detect SNP in MDD at p<0.05 | | | | |  | Empirically estimated effect on MDD in PGC | |
|  | Allele Frequency | Beta | Variance explained |  | Fully causal | |  | Quarter causal | |  |  |  |
| SNP |  |  |  |  | Var MDD (%) | power |  | Var MDD (%) | power |  | Beta MDD | p-value |
| Exposure tested Vitamin D | | | | | | | | | | | | |
| rs10741657 | 0.40 | 0.031 | 2.5e-01 |  | 1.2e-02 | 1.00 |  | 3.1e-03 | 0.89 |  | -0.007 | 0.139 |
| rs10745742 | 0.40 | 0.016 | 7.1e-02 |  | 3.5e-03 | 0.92 |  | 8.9e-04 | 0.40 |  | 0.001 | 0.866 |
| rs12785878 | 0.75 | 0.036 | 3.4e-01 |  | 1.7e-02 | 1.00 |  | 4.3e-03 | 0.96 |  | -0.001 | 0.820 |
| rs17216707 | 0.79 | 0.026 | 1.2e-01 |  | 6.0e-03 | 0.99 |  | 1.5e-03 | 0.60 |  | 0.003 | 0.616 |
| rs3755967 | 0.28 | -0.089 | 1.9e+00 |  | 9.5e-02 | 1.00 |  | 2.4e-02 | 1.00 |  | 0.002 | 0.705 |
| rs8018720 | 0.82 | -0.017 | 4.2e-02 |  | 2.1e-03 | 0.74 |  | 5.3e-04 | 0.26 |  | -0.002 | 0.724 |
| Exposure tested N3-PUFAs | | | | | | | | | | | | |
| rs1077835 | 0.25 | 0.089 | 4.8e-02 |  | 1.9e-03 | 0.70 |  | 4.8e-04 | 0.24 |  | -0.002 | 0.763 |
| rs11604424 | 0.76 | -0.090 | 5.0e-02 |  | 2.0e-03 | 0.73 |  | 5.0e-04 | 0.25 |  | 0.003 | 0.600 |
| rs1260326 | 0.64 | -0.097 | 7.3e-02 |  | 2.9e-03 | 0.87 |  | 7.3e-04 | 0.34 |  | 0.006 | 0.230 |
| rs143988316 | 0.07 | -0.171 | 6.2e-02 |  | 2.5e-03 | 0.82 |  | 6.2e-04 | 0.30 |  | -0.010 | 0.242 |
| rs150617279 | 0.11 | -0.121 | 4.3e-02 |  | 1.7e-03 | 0.66 |  | 4.3e-04 | 0.22 |  | -0.021 | 0.037 |
| rs174546 | 0.40 | -0.154 | 1.9e-01 |  | 7.7e-03 | 1.00 |  | 1.9e-03 | 0.71 |  | 0.023 | 7.3e-6 |
| rs183688424 | 0.03 | -0.159 | 2.2e-02 |  | 8.9e-04 | 0.40 |  | 2.2e-04 | 0.13 |  | 0.036 | 0.274 |

**References**

1. Mbarek H, Milaneschi Y, Hottenga JJ, et al. Genome-Wide Significance for PCLO as a Gene for Major Depressive Disorder. *Twin Res Hum Genet*. 2017;20(4):267-270. doi:10.1017/thg.2017.30.

2. Francioli LC, Menelaou A, Pulit SL, et al. Whole-genome sequence variation, population structure and demographic history of the Dutch population. *Nat Genet*. 2014;46(8):818-825. doi:10.1038/ng.3021.

3. Das S, Forer L, Schönherr S, et al. Next-generation genotype imputation service and methods. *Nat Genet*. 2016;48(10):1284-1287. doi:10.1038/ng.3656.

4. Yang J, Lee SH, Goddard ME, Visscher PM. GCTA: A tool for genome-wide complex trait analysis. *Am J Hum Genet*. 2011;88(1):76-82. doi:10.1016/j.ajhg.2010.11.011.

5. Kettunen J, Demirkan A, Würtz P, et al. Genome-wide study for circulating metabolites identifies 62 loci and reveals novel systemic effects of LPA. *Nat Commun*. 2016;7:11122. doi:10.1038/ncomms11122.

6. Watanabe K, Taskesen E, Bochoven A van, Posthuma D. FUMA: Functional mapping and annotation of genetic associations. *Nat Commun*. 2017;8:1826. doi:10.1101/110023.

7. Purcell SM, Chang CC, Chow CC, Tellier LC, Lee JJ, Vattikuti S. Second-generation PLINK: rising to the challenge of larger and richer datasets. *Gigascience*. 2015;4(1):1-16. doi:10.1186/s13742-015-0047-8.

8. Jiang X, O’Reilly PF, Aschard H, et al. Genome-wide association study in 79,366 European-ancestry individuals informs the genetic architecture of 25-hydroxyvitamin D levels. *Nat Commun*. 2018;9(1):260. doi:10.1038/s41467-017-02662-2.

9. Major Depressive Disorder Working Group of the Psychiatric Genomics Consortium. Genome-wide association analyses identify 44 risk variants and refine the genetic architecture of major depressive disorder. *Nat Genet*. 2018. doi:https://doi.org/10.1101/167577.

10. Vilhjálmsson BJ, Yang J, Finucane HK, et al. Modeling Linkage Disequilibrium Increases Accuracy of Polygenic Risk Scores. *Am J Hum Genet*. 2015;97(4):576-592. doi:10.1016/j.ajhg.2015.09.001.

11. Milaneschi Y, Lamers F, Peyrot WJ, et al. Genetic Association of Major Depression With Atypical Features and Obesity-Related Immunometabolic Dysregulations. *JAMA psychiatry*. 2017. doi:10.1001/jamapsychiatry.2017.3016.

12. Lee SH, Goddard ME, Wray NR, Visscher PM. A Better Coefficient of Determination for Genetic Profile Analysis. *Genet Epidemiol*. 2012;36(3):214-224. doi:10.1002/gepi.21614.

13. Hemani G, Zheng J, Elsworth B, et al. The MR-Base platform supports systematic causal inference across the human phenome. *Elife*. 2018;7:e34408. doi:10.7554/eLife.34408.

14. Watanabe K, Stringer S, Frei O, et al. A global overview of pleiotropy and genetic architecture in complex traits. *bioRxiv*. 2018. doi:10.1101/500090.

15. Bowden J, Smith GD, Burgess S. Mendelian randomization with invalid instruments: Effect estimation and bias detection through Egger regression. *Int J Epidemiol*. 2015;44(2):512-525. doi:10.1093/ije/dyv080.

16. Okbay A, Baselmans BML, De Neve J-E, et al. Genetic variants associated with subjective well-being, depressive symptoms, and neuroticism identified through genome-wide analyses. *Nat Genet*. 2016;48(6):1-13. doi:10.1038/ng.3552.

17. Peyrot WJ, Boomsma DI, Penninx BWJH, Wray NR. Disease and Polygenic Architecture: Avoid Trio Design and Appropriately Account for Unscreened Control Subjects for Common Disease. *Am J Hum Genet*. 2016;98(2):382-391. doi:10.1016/j.ajhg.2015.12.017.

**Data and software availability**

GWAS summary statistics used in the present analyses have been retrieved at the following URLs:

25-OH-D: <https://drive.google.com/drive/folders/0BzYDtCo_doHJRFRKR0ltZHZWZjQ> ; n3-PUFA: <http://www.computationalmedicine.fi/data#NMR_GWAS> ;

MDD: <https://www.med.unc.edu/pgc/results-and-downloads> (the statistics publicly available are based on a GWAS not including 23andMe data, access to which is restricted by a Data Transfer Agreement).

NESDA data could be requested at <https://www.nesda.nl/nesda-english/> ;NESDA genotype data were also previously included in the larger GAIN-MDD GWAS dataset already available at dbGaP (Study Accession: phs000020.v2.p1).

PLINK <https://www.cog-genomics.org/plink/1.9/>

LDpred <https://github.com/bvilhjal/ldpred>

MR-Base for two-samples MR <https://mrcieu.github.io/TwoSampleMR/>

mRnd, power calculator for MR <http://cnsgenomics.com/shiny/mRnd/>

FUMA <http://fuma.ctglab.nl/>

LDLink <https://ldlink.nci.nih.gov/>

ATLAS GWAS <http://atlas.ctglab.nl/>

CGTA <https://cnsgenomics.com/software/gcta/#Overview>
